# Education and microfinance: an alternative approach to the empowerment of the poor people in Indonesia

**DOI:** 10.1186/s40064-015-0995-6

**Published:** 2015-06-04

**Authors:** Rizali Hadi, Uyu Wahyudin, Jajat S Ardiwinata, Wamaungo Juma Abdu

**Affiliations:** Universitas Lambung Mangkurat (UNLAM), Jalan Brigjen H. Hasan Basri, P.O. Box 219, Banjarmasin, 70123 South Kalimantan Indonesia; Department of Nonformal and Continuing Education, Faculty of Education, Universitas Pendidikan Indonesia, Jalan Dr. Setiabudhi No.229, Bandung, Jawa Barat Indonesia; Center for Research and Community Service (LPPM), Universitas Pendidikan Indonesia, Jalan Dr. Setiabudhi No.229, Bandung, Jawa Barat Indonesia

**Keywords:** Alternative approach, Education, Education and microfinance combined, Empowerment, Indonesia, Microfinance, and poor

## Abstract

There is good reason to combine education with microcredit for poverty alleviation in the poor communities of the developing world, including in Indonesia. Poverty is dangerous, it deprives people of their right to education, their right to good health, their right to freedom of speech, their right to democracy, their right to financial services and of course their right to knowledge enhancement, which are all crucial to living a better life. We must therefore, provide services beyond, credits for the poor. In this case, education should be included to each and every development agenda for the poor since it is key to any positive change and sustainable development of people. If well planned and well integrated within the microcredit services, education can serve a good purpose in poverty alleviation. This paper describes how education and microfinance have been used in combination to alleviate poverty in Indonesia, especially in the areas studied. The study uses a multi-cases approach to examine the purposively selected *baitul maal tamwil* (BMTs) organisations, which are sharia based semiformal microfinance institutions regarded to be among those few integrating education with their financial services.

## Introduction

Indonesia is a huge country, divided with Islands, and with a diversity of language(s), religion(s), and culture and with a wide geographical location. The country faces a lot of the development challenges which include poverty and incompetent human resources moreso in the informal sector where the majority of the people are poor. This makes education and poverty alleviation some of the national development agenda(s). To eliminate poverty, microfinance has been in place for a number of years. Bank Rakyat Indonesia (BRI) is proud of its success story in providing microcredits to the grassroot communities for over one hundred years now. According to Seibel “the sector now comprises some 6,300 formal and 47,200 semiformal microfinance outlets, serving about 47 million deposit accounts and 32 million loan accounts. Among them, the BRI Units (formerly unit *desa*) account for 80% of microsavings balances and 54% of microloans outstanding” (Seibel 2005).

However, despite the successful stories on microfinance, studies continue to show that poverty is still a big problem for the developing world. The rate at which it affects the poor is alarming. Ferreira and Ravallion (2008) note that one billion people subsisting on per capita incomes less than one dollar per day live in developing countries. Sachs (2005) establishes that more than eight million people around the world die each year because they are poor to stay alive.

**Figure 1:**
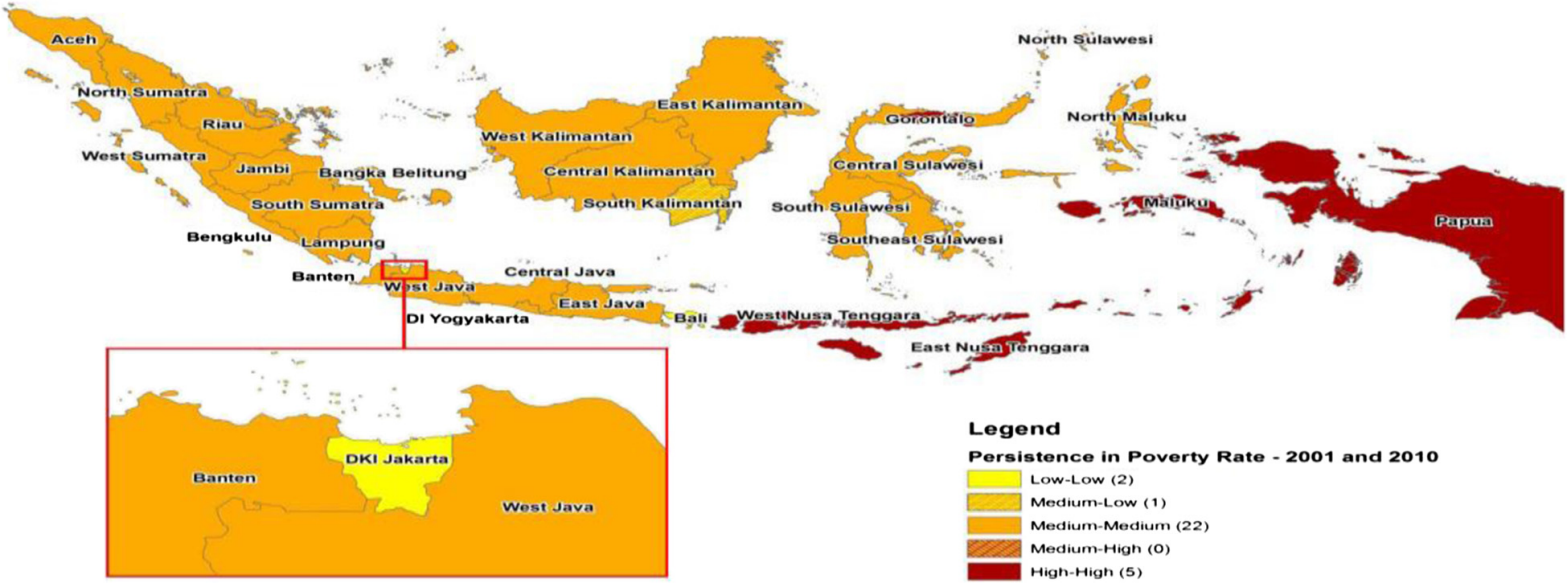
Persistence of Poverty Rates (2001 and 2010). Source: Adapted from Miranti et al. (2013), “Trends in Poverty and Inequality in Decentralising Indonesia”, OECD Social, Employment and Migration Working Papers, No. 148, OECD Publishing.

In Indonesia, the poor people remain highly vulnerable to changes in economic, social and political conditions and natural disasters which occur in the different regions (Moeliono, et al 2007) of the country. According to the National Forum Coordination Education for All (2005) Indonesia’s economy experienced a negative growth in the past and its growth slowed for some years. In cities, approximately 18 percent of the populations are said to be poor, representing some 20 million people. As the country urbanises, this number is expected to increase, surpassing rural poverty by the year 2020 (Policy Note: Indonesia 2012). The Figure 1 below illustrates persistence and the spread of poverty rates across Indonesia:

As shown in the figure above, classifications are made based on the standard deviation from the average, in which the 30 provinces are categorised as either low, medium or high (Miranti et al. 2013). Indonesia defines the benchmark for poverty as a situation where a person can or cannot fulfill the cost of basic needs of food items with an intake of 2100 calories per day and also non-food items (Miranti et al. 2013). To eliminate poverty several steps have been undertaken ‘since the beginning of the reform period’.

Change of government in 1997/1998 led to reforms in the programmes which were intended to fight poverty across the country. These reforms took place after the evaluation of the prior centralised approaches to poverty alleviation and public consultations with government, universities, NGOs, donor organisations, economic players and the poor communities (Andrianto 2006). This indicated that the centralised approach was out of date and could no longer serve community needs or individual interests. It meant that, time had come to stop considering the poor communities as objects of development, but rather to be considered active participants to development (Andrianto 2006) and a solution to poverty alleviation. The grassroot community organisations, religious organisations and also other non-government organisations were established specifically to bring poverty alleviation services near to the grassroot communities. Some of these organisations have been and continue to offer education with credit programmes and among them is MiSyikat and BMT Khalifa sharia based semiformal microfinance institutions.

Education and microfinance have been combined because education alone as an empowerment tool has failed in some areas to support the country’s efforts to empowerment of the poor. For instance, Ki Supriyoko as reported by Rulistia (2009) points out that “one in every eight Indonesians is still illiterate”. This shows that Indonesia’s education performance is still poor compared to other countries in the region (Rulistia 2009). While according to Policy Note: Indonesia (2012) in cities, around 18 percent of the populations are poor and this number is predicted to increase surpassing rural poverty by the year 2020. This implies that besides the challenges education faces, the microfinance sector also faces some challenges in its fight against poverty. Perhaps a combination of the two can serve a fruitfull purpose to economic empowerment and knowledge enhancement of the poor at the grassroot.

Thus, innovative approaches including microfinance have surfaced as either adoptions from foreign states or local innovations which have emerged as a response to either the education or microfinance failures. One of such approaches is the education and microfinance approach which combines training for knowledge enhancement and skills development with a financial component for grassroot community empowerment. This is mainly done by the semiformal, nonformal and informal microcredit organisations who believe that the grassroot communities have not benefited from either government efforts and or the formal banking institutions, *i.e.*, conventional banks to attain financial empowerment.

In regard to this point, studies and news reports continue to show that many families are not benefiting from national economic growth and that fifty percent of Indonesia’s population is still poor, hovering around the poverty line, living on less than US $2 per day (Handayani 2012). Handayani further reports that the Center for Welfare Studies mentions that the number of poor people in Indonesia increased by 6.7 percent over the last three years, totalling to over 43.1 million people (2012). This very report also shows that the number of Indonesians living in extreme poverty was 40.36 million in 2008, 44.83 million in 2009, and 43.07 million in 2010. However, in 2011, Indonesia experienced a decline in poverty rates from 12.49 percent in March, but it slided to 12.36 percent in September (Handayani 2012).

However, though there reports which have kept showing widespread poverty in Indonesia on the one hand, there is also empirical researches which have also continued to show success in Indonesia’s microfinance sector. This is evidenced by the fact that Indonesia has managed to attain positively on its Millennium Development Goals (MDGs) agenda. This may be the reason as to why, the meeting of the High Level Panel of Eminent Persons (HLPEP) was held in Bali from 25-27 March of 2013 (Karang 2013). In other word, Indonesia has registered success in relation to its poverty alleviation programmes compared to other developing countries. This is marked by a letter of recognition which was received by the President of Indonesia in 2012. Karang (2013) reports, “President Susilo Bambang Yudhoyono received a letter of recognition from the international microfinance community for his outstanding achievement in microcredit programmes, which have been considered successful in reducing poverty and unemployment”.

It is this success story which motivated us “to conduct a study that aims to describe Indonesia’s education and microfinance model in poverty alleviation and empowerment of the poor people”. In specific, we aimed to establish an alternative approach that could help to empower the poor members of society within the developing world and of course in Indonesia.

### Literature review

Today, microfinance has grown substantially with the hope of helping to reduce poverty (Valdivia et al. 2008). The industry is now a household term with frequent articles in the media about its growth, innovation, and its impact (Ledgerwood 2013) on the peoples’ lives around the glob. However, though the microfinance industry has registered success, it has also registered some challenges and weaknesses.

This has led to debates among academics and the microfinance providers who have slowly started to accept that just ‘micro-credits’ are no longer enough to improve the poor people’s living standards. The focus is no longer only on credit for investment in microenterprises (Ledgerwood 2013), but there is a strong trend to innovations which have led to a combination of microfinance with non-financial services, including business training (Dunford 2002; Valdivia et al. 2008) which is an education aspect. The microfinance institutions have come to discover that capital, knowledge, and opportunity are the three key items that will help to empower the poor (Saleha 2009).

The World Bank mentions that the best way to help people climb out of poverty is to give them an education (Narayan and Petesch 2007). Education is the most effective tool for human development (Wamaungo, 2011). Through education, people can learn what is necessary for life. Education can help to strengthen or improve the prevailing shift from microcredit to microfinance (Ledgerwood 2013), and to financial inclusion and financial literacy (Wamaungo 2014). In this sense combining education and microfinance services for human prosperity is of great importance. Education and easy access to credit services are fundamental elements in the support of the microfinance programmes. Education and microfinance have a fundamental role to play in personal and social development. Education is one of the principle means available to foster a deeper and more harmonious form of human development and thereby to reduce poverty, exclusion, ignorance, oppression and war (Delors et al. 1996).

The UNDP (2008) mentions that lack of access to essential resources goes beyond financial hardship to affect people’s health, education, security and opportunities for political participation”. Poverty is traditionally viewed as lack of income, assets and the resources but recent studies recognise that it includes issues related to dignity and autonomy (Cagatay 1998; Hussain and Mahmood 2012) which can be attained through education. In other words, education activities are important any programme intended for human empowerment.

Dignity and autonomy are reflected in Maslow’s work on Hierarchy of needs. These needs can be achieved through education. Education is needed as a vehicle for change and development (Wamaungo 2011). It is a crucial factor for all aspects of development and poverty alleviation. It is one of the effective tools in poverty elimination (Leowarin 2010). In Indonesia, microfinance has its own success story to tell. The institutions of microfinance have been categorised into: semiformal, formal, nonformal and Informal institutions. Microfinance has generated worldwide enthusiasm as a potential answer to economic development and poverty reduction.

Several studies have been carried out about microfinance in Indonesia, including banks and non-banks but most of them are focused on the availability of credit services (Wamaungo 2014. In this research, the objective is to establish an education and microfinance combined empowerment approach that can help to improve the condition of the poor adults in society. The goal is to discover and promote the appropriate education and microfinance combined approach that can help to empower the microcredit clients with a low income background.

Currently, the poor face the challenges which include hunger, diseases, waste of life (Sachs 2005), economic crisis, increased poverty, malnutrition, consistent illitracy among others. Amidst all these unlimited challenges or problems, education remains important. Its function is to empower both the haves and the have nots at all times. Sachs (2005) describes this as crossing human with financial capital, which entails a new strategy and planning for the poor. The more a country transforms the more vital education becomes. This does not mean that education is ‘everything’, but every development programme will always require some kind of awareness creation which is one form of education.

If the poor are well educated or well trained or equipped with the neccessary skills, can attain a better socio-economic status and are able to enjoy better health, employment prospects, and of course can contribute to the increase in national income (OECD 2012). This is probably why, the UNDP’s first Human Development Report (HDR) of 1990 focused on human empowerment by proposing poverty alleviation, and the improvement of human welfare (Delors et al. 1996) as foundational components for both human empowerment and national development. This is also supported by the Millennium Development Goals (MDGs) among the goals is a mention of “Universal Basic Education” and the “Eradication of Extreme Poverty” by the year 2015.

According to Ginzberg’s human economy theory, it is noticed that for change to occur, it needs an understanding and a guide on the course of action. Ginzberg believes that it is education and industrialization that can assist to bring about this change. Second, Ginzberg (1971) also notes that the rate at which capital can be absorbed is determined in large measure by the manpower resources available in the higher reaches of the economy that is, in the government and the business sectors-as well as by the number of skilled workers and technicians distributed throughout other branches of the economy. A country’s know-how only requires to put people and other resources together to make products desired (Ginzberg 1971). Third, Ginzberg is of the assumption that, human resource is the only live resource, and its potential is without limit (Ginzberg 1971). In other words, human beings are just limited by the prevailing circumstances, but if properly empowered and adequately equipped can do the impossible. This is the case with people of poor skills; they face a much greater risk of experiencing economic disadvantage and a higher likelihood of unemployment and dependency on social benefits (OECD 2012) or no social benefits at all since it is not common in the developing countries.

The human capital theory propounds that education or training raises the productivity of workers by imparting useful knowledge and skills, hence raising workers’ future income by increasing their lifetime earnings (Becker 1964). The best way to empower people is by giving them education. Education is considered the most effective tool for human transformation (Wamaungo, 2011). Through Education, people can learn what is necessary for life. The UNESCO (2001) handbook on “*Effective Implementation of Continuing Education at the Grass-roots*” mentions that: a) education is a social activity, which involves people working cooperatively and serving each other, and b) education empowers people- it is a tool for changing our society. Thus, the empowerment of people is an important result of education.

### Research methodology

In this study, a multiple cases approach was used. This approach is used to examine several cases ranging from two to as many as possible cases (Creswell 2008; Wamaungo, 2014). With this approach, the researchers scouted for possible places and people (Bogdan and Biklen 1982) that were considered as the subjects of the study (Wamaungo, 2014). Two semiformal credit and savings organisations were involved.

Based on the preliminary study and the review of literature, it was established that one of the factors that contribute to the success of Indonesia’s microfinance sector is the local creativity and innovations of the local actors in the sector. Among the innovations is a combination of education with microfinance.

During the preliminary study, we established that many activities combining education and microfinance services are in place, but little is known about their existence because: 1) Indonesia’s financial service policy limits the financial service sector, and 2) It is true that firms combining education and microfinance are either small micro-companies, and or non-government organisations (Wamaungo, 2014), therefore, little attention is paid to their services and or even their existence is little known. Though the two samples studied cannot represent the whole Indonesia, the authors have tried to establish a clear overview for the whole empowerment process using this approach.

### Location and subjects of the study

No research takes place in a vacuum, every research takes place in society. It is by this way that each and every researcher can obtained the required information to a study (Wamaungo 2014). This study took place in two semiformal microfinance institutions both located in Bandung city. Bandung is the capital city of West Java province in Indonesia (Wamaungo 2011), the country’s third largest city, and second largest metropolitan area in Indonesia with a population of 2.4 million (Badan Pusat Statistik 2007).

In this study, two cases were examined, they included: BMT Khalifa and MiSykat community empowerment programme of *Darul Tahuhiid*. The two organisations are both located in Bandung city. BMT Khalifa: is a community based sharia microfinance institution founded in 2007. It is dedicated to serving and assisting the grass-root communities. Microfinance *Syariah Berbasis Masyarakat*: which is shortened as *MiSykat* is translated to English as “Community Based Sharia Microfinance”. It is an empowerment programme for the poor. This programme categorises the poor in two groups: the poor people (they own some property but they cannot afford most of their basic needs), and the extremely poor (this group of people hardly owns anything except life).

### Procedure of the study

Procedure of a study in a qualitative research may have varying forms and degrees of specificity depending on whether or not the researcher has been able to undertake any pre-proposal fieldwork (Gay et al. 2006; Wamaungo 2014). On the same hand Fraenkel and Wallen state that procedure of the study is what a researcher will do (what, when, where, how and with whom) from beginning to the end (Wamaungo, 2014). Creswell (2008) advises us to design a qualitative data collection procedure for our educational project. He further states that we should determine the people and research sites we will study and the type of purposeful sampling we will use (Wamaungo, 2014).

The first step in the process of collecting qualitative data is to identify the people and places you plan to study. This involves determining whether a researcher will study individuals or entire organizations (*e.g.*, schools, institutions) or some combination. If a researcher selects either individuals or organizations, he or she needs to decide what type of people or organizations he or she will actually study and how many he or she will need for his or her research. These decisions require that a researcher to decide on a unit of analysis, the group and individuals he or she will study, the procedure for selecting these individuals, and assessing the numbers of people needed for his or her data analysis (Creswell 2008). This will also include the unit of analysis whereby a researcher will have to identify the supply of information which will answer the research questions.

### Data collection techniques

Data collection is the process of gathering and measuring information on variables of interest, in an established systematic fashion that enables a researcher to answer stated research questions, test hypotheses, and evaluate outcomes. The data collection component of research is common to all fields of study. The typical qualitative study involves a number of different data collection strategies and although all options are open, some strategies are used more often than others (Gay et al. 2006). The data collection techniques we used were observation, both non-directive and unstructured interviews. We also studied documents and made audio visual analysises.

Interview: The interview technique was mainly used on programmes which had integrated education in microfinance for community building and empowerment in BMT Khalifa and Misykat. We sought to describe the meanings of central themes in the real life situation of the subjects. The main task in interviewing is to understand the meaning of what the interviewees say. A qualitative research interview seeks to cover both a factual and a meaning level, though it is usually more difficult to interview on a meaning level. (Kvale 1996) On the same hand interviews are particularly useful for getting the story behind a participant’s experiences (McNamara 1999).

Group Discussions: We gathered information through focus group discussions. According to Neuman (2003) a focus group is a special qualitative research technique in which people are informally interviewed in a group discussion setting. Focus groups must have moderators who should be nondirective and manage to facilitate free and open discussion by all group members. The focus group discussions comprised of 11 people. They came from the two organisations though conducted on different occasions. The focus group discussions were conducted six (6) times. The researcher wanted to establish the views of the clients about the empowerment programmes of the two organisations.

Observation: as a technique for data collection is about acquiring the data or information through watching the object of research. The researchers used this technique to observe the education and microfinance programme in its natural setting in both the two organizations. While in the field, the researchers participated in two ways, as active observers and in some cases as passive observers. For instance, during the trainings, the researchers were more of passive observers and during discussions, the researchers could intervene. During the process of observation, the researchers also used a guide; this guide was more specific and clear on what to be done.

### Findings

It has been discovered that in the effort to integrate education with microcredit, the two organisations run short term curriculums and follow-up schedule curriculums. Their short term curriculums consist of the following in common:

Phase One: Basics to Financial Management

- Economic empowerment;
- Step by step saving skills;
- Understanding savings and credit
- Assessment

Phase Two: Religious Financial Education

- Islamic loans
- Principles of working in partnership
- Signing agreements
- The importance of debt payment.
- Avoiding failure to pay your debt
- Simple methods and techniques in book keeping
- Assessment

Phase Three: Building a Strong Household Economy

- Religion and its financial principles for married people
- Family trust and responsibility
- Faith, prosperity and harmony in a family
- Understanding sins in relation to the use of family income

With the above curriculum content, it is evident that the two organisations are combining education in their financial services. Though they may not be calling such programmes education (instead using names like couching, and religious tutorials). We categorised this as education since there learning objectives, schedule and a stated time frame for the programmes.

**Figure 2:**
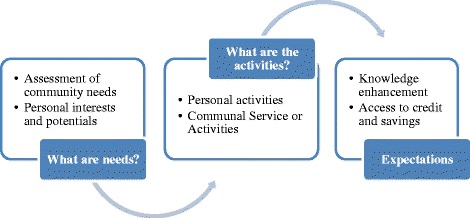
An Illustration of Individual Assessment, Identification of Learning Needs and Expected Outcomes.

As seen in the schedule, it is established that these organisation offer trainings on financial management basics, religious principles to financial management and household income generation projects. The education activities are integrated with credit services to help equip the clients with necessary entrepreneurship knowledge and business skills required for life improvement. Individual learning needs are catered for as illustrate in Figure 2 below:

This figure describes an important aspect that may be one of the factors which have led to the success of the education and microfinance empowerment approach for the poor. It reveals that programmes developed are based on communal and personal needs of the participating community members.

**Figure 3:**
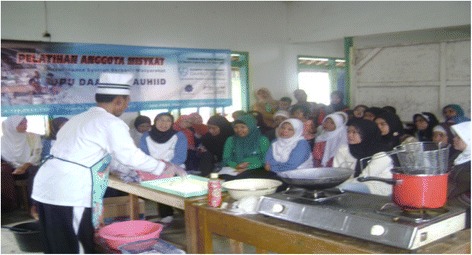
Shows one the Trainings intended to enhance the Poor’s Knowledge and Business Skills for Sustainability.

**Figure 4:**
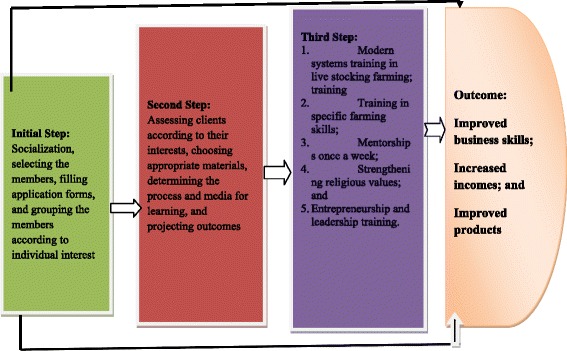
Common Steps followed through to Empowerment of the Poor: Combinig MiSykat and BMT Khalifa’s Activities.

The findings also continue to show that education and microfinance could support entrepreneurship knowledge enhancement and skills development of the poor through training and mentorship. This is illustrated as in Figure 3 below:

This is an illustration of a training on cooking for the small scale food industry known as ‘*warung’*. The *warung* owners are trained to cook and also to manage their finances. This is one of the activities conducted in the process of integrating education with microcredit for the empowerment the poor. The participants are equipped with the neccessary entrepreneurship knowledge and skills needed to manage their daily affairs (in this case the *warung makan* business). There are three steps followed, as shown in the Figure 4 below:

Education with microfinance approach is expected to lead to a productive economy; an economy that is supported by all citizens, whose basis is partcicipatory development or inclusive development. In other words, all citizens have equal opportunities to life improvement. It means growth and transformation is a communal thing for all members.

## Discussion

The clients from the two organisations have varying experiences which led them join these two institutions (i.e., MiSyikat and BMT Khalifa). Some of them share experiences and others have different life stories to tell. However, the most common thing is that, they all have reasons for joining. For instance, most of them are happy due to the services provided by these organisations. The orientations of the education with microdit services aim to empower the clients. By providing education to the clients, they are in position to manage their businesses without difficulties. Besides, providing credit and savings is a good service for the poor clients who have no collateral to access finances from formal institutions.

The education element is about financial education. The aspects covered in financial education include: finance management, financial literacy, the importance of savings and how to save *etc.*

Education is considered the most effective tool for human transformation. Without education, the clients may appear prosperous, but they cannot tell where shortfalls are coming from in their businesses. Second, to develop communities they should not only depend on social assistance funds but they should be encouraged to work. The World Bank has argued that the best way to help people out poverty is to give them education. Education is considered the most effective tool for human transformation.

Through Education, people can learn what is necessary for life. The UNESCO (2001) handbook on “Effective Implementation of Continuing Education at the Grass-roots” mentions that: a) education is a social activity, which involves people working cooperatively and serving each other, and b) education empowers people- it is a tool for changing our society. Thus, the empowerment of people is an important result of education. The capacity of the workforce determines the country’s abilities to generate growth and wealth (Osmankovic et al. 2011). Ginzberg believes that it is education and industrialization that can assist to bring about this change. Second, Ginzberg (1971) is of the view that the rate at which capital can be absorbed is determined in large measure by the manpower resources available and the number of skilled workers and technicians distributed throughout other branches of the economy. A country’s know-how only requires to put people and other resources together to make products desired (Ginzberg 1971). Ginzberg is of the assumption that, human resource is the only live resource, and its potential is without limit (Ginzberg 1971). In other words, human beings are just limited by the prevailing circumstances, but if properly empowered and adequately equipped can do the impossible. This is the case with the poor.

The main objective of this combined approach is to empower the poor and also to promote self-reliance among the members of the grassroot communities. By setting clear objectives, it helps to establish clear programmes, such as poor client oriented empowerment programme. According to the findings, it has been discovered that poor client oriented empowerment programme can easily lead to change and hence prosperity- this is because the clients are encouraged to establish small scale business enterprises which are based on individual needs and interest. Thus, each and every participant has individual inner motivation and power to develop.

This research shows that people are just limited by the existing structures and that these structures are man-made. It means, for any positive change, there is need to manage the whole process or structure of human resources in order to reach the goal of development. MiSykat uses a mentorship system, which is semi-structured, intensive and comprehensive to help its member get out of the cage (Wamaungo 2014). Efforts are made to help the very poor to become self-empowered through this mentorship process.

Some of the activities include: small business enterprises encouragement programme, alms for the poor and the very poor, encouraging clients to live in one place than changing places of stay, while BMT Khalifa is of the assumption that to develop poor communities they should not only be given social assistance funds but they should be encouraged to work. They should be motivated to reveal their real potential.

Another way to help the poor is through knowledge enhancement. If the poor are well educated (trained or their skills enhanced), can attain a better socio-economic status and are able to enjoy better health, employment prospects, and of course can contribute to the increase in national income. The UNDP’s first Human Development Report (HDR) of 1990 focused on human empowerment by proposing poverty alleviation and the improvement of human welfare (Delors et al. 1996) as foundational components for both human empowerment and national development. This is also supported by the Millennium Development Goals (MDGs) among the goals is: “Universal Basic Education” and the “Eradication of Extreme Poverty” by the year 2015.

Indonesian government emphasises human development as the key to development. To develop a country’s human quality is fundamental. Marshall writes that the most valuable of all capital is that invested in human beings (Becker 1975). Human capital investment is a process under which human beings are developed in knowledge and skills to serve production purpose. Individual production is of importance to the microfinance clients since they are employers of themselves.

## Conclusions

Education activities if well integrated with credit services can empower the clients by helping them develop the necessary entrepreneurship knowledge and business skills required to operate in daily life activities. With this approach, each individual’s learning needs are catered for and empower the poor clients to run sustainable businesses and above all, these clients are able to pay back loans taken.

Even those who could not be trusted have now become responsible borrowers either of MiSyikat or BMT Khalifa. The approach has proved that even the poor are able to change their condition. This means that whoever, intends to apply the education with microcredit approach should go slow until clients have attained a certain level of sustainability.

In conclusion, education and microfinance are combined because each of the activities can ‘no-longer stand alone’. In other words, nonfinancial innovations such as education are highly needed to support the microfinance industry in knowledge enhancement, while credits are needed to offer financial support to the poor people of the community.
